# Keratin 7 expression in hepatic cholestatic diseases

**DOI:** 10.1007/s00428-021-03152-z

**Published:** 2021-07-27

**Authors:** S. Sakellariou, C. Michaelides, T. Voulgaris, J. Vlachogiannakos, E. Manesis, D. G. Tiniakos, I. Delladetsima

**Affiliations:** 1 1st Department of Pathology, Medical School, Laiko General Hospital, National and Kapodistrian University of Athens, 75 Mikras Asias str, 11527 Athens, Greece; 2 Academic Department of Gastroenterology and Hepatology, Laiko General Hospital, National and Kapodistrian University of Athens, 17 Agiou Thoma str, 11527 Athens, Greece; 3 Liver Unit, Euroclinic, 7-9 Athanasiadou str, 11521 Athens, Greece; 4 Department of Pathology, Aretaieion Hospital, National and Kapodistrian University of Athens, 76 Vasilissis Sofias Ave, 11528 Athens, Greece; 5 Translational & Clinical Research Institute, Faculty of Medical Sciences, Newcastle University, Framlington Place, Newcastle upon Tyne, NE2 4HH UK

**Keywords:** Keratin 7, Cholestasis, Ductular reaction, Bile duct obstruction, Primary sclerosing cholangitis, Primary biliary cholangitis, Acute hepatitis, Fibrosis, GGT, Transaminase

## Abstract

We evaluated keratin 7 (K7) hepatocellular expression in 92 patients with common types of acute and chronic cholestatic diseases caused by bile duct obstruction/destruction or parenchymal lesions [acute hepatitis (*n*=20), mixed/pure cholestasis (*n*=16), primary biliary cholangitis-PBC (*n*=35), primary sclerosing cholangitis-PSC (*n*=10), vanishing bile duct syndrome (*n*=3), complete large bile duct obstruction due to space-occupying lesions (*n*=8)]. K7 immunohistochemical hepatocellular expression and ductular reaction (DR) were semi-quantitatively assessed. Results were correlated with liver enzyme serum levels, cholestasis type, histological features, hepatocellular Ki67 labelling index (LI) and HepPar1 expression. Hepatocellular K7 expression was detected in 87% (81/92) cases and in all cholestatic disease types with lowest incidence in pure/mixed cholestasis and highest in incomplete bile duct obstruction (iBDO), reaching 100% in PSC. K7-positive hepatocytes had low Ki67 LI (0-5%) retaining HepPar1 expression, irrespective of disease type. PSC cases had high K7 hepatocellular expression even with intact bile ducts, a feature that may aid differential diagnosis of cholestatic syndromes. K7 hepatocellular expression significantly correlated with cholestasis type, bile duct loss and fibrosis stage. It was higher in milder acute cholestatic hepatitis showing inverse correlation with hepatocyte proliferation and serum transaminase levels. In iBDO, younger age independently correlated with high K7 expression, while serum GGT levels showed a nearly significant correlation. Correlation with DR findings implied that K7-positive hepatocytes may result through metaplasia. In conclusion, K7 hepatocellular expression is a sensitive though non-specific marker of cholestasis. It may represent a cytoprotective reaction of resting hepatocytes in cholestasis of longer duration especially in younger patients.

**Supplementary Information:**

The online version contains supplementary material available at 10.1007/s00428-021-03152-z.

## Introduction

In normal liver, keratin 7 (K7) belongs to the spectrum of keratins that define the immunophenotype of cholangiocytes. Keratin 7 is also expressed in hepatoblasts and hepatic progenitor cells (HPC) but not expressed in normal hepatocytes constituting a marker of liver cell immaturity [1, 2]. K7-positive hepatocytes and bile ductules are considered maturation steps in the bipotent HPC differentiation pathway. In addition, Κ7 expression by intermediate and mature-appearing hepatocytes may also represent steps in hepatocytic transdifferentiation to cholangiocytes (biliary metaplasia) [3, 4] or dedifferentiation to HPC [5].

K7-positive hepatocytes singly or in groups are encountered in a variety of liver diseases, including viral and autoimmune hepatitis (AIH), alcoholic and non-alcoholic steatohepatitis, cirrhosis of any aetiology [6–10] as well as in massive and submassive hepatic necrosis [11, 12]. Moreover, increased numbers of K7-positive hepatocytes with acinar zone 1 predilection have been associated with cholestatic injury, while their detection in zone 3 has recently been associated with ischemia supporting different pathogenetic links [1, 13–17].

Cholestasis is characterised by partial or complete interruption of normal bile flow. The underlying causes are bile duct obstruction/destruction or malfunction in bile production and secretion caused by hepatocyte injury [18, 19]. The presence of K7-positive hepatocytes is considered a morphological sign of chronic cholestasis [10, 13]. They are primarily located periportally or periseptally and are thought to represent an early sign of biliary metaplasia leading to bile ductule formation described as ductular reaction type 2A by Desmet [20]. Within this context, K7-positive hepatocytes constitute an additional feature in chronic biliary diseases, such as primary biliary and sclerosing cholangitis, as well as in drug induced parenchymal injury [2, 10, 13, 14]. The use of Κ7 has been proposed to aid diagnosis and assessment of disease evolution [2, 13, 21, 22]. However, the presence, extent and distribution pattern of K7 hepatocyte expression in relation to the cause and severity of the underlying cholestatic disease have not been systematically examined to date.

In the present study, we evaluated aberrant K7 expression of hepatocytes in relation to histological and biochemical parameters in the most frequent cholestatic diseases. Our aim was to clarify the significance of K7 immunostain in defining the type of cholestasis within the context of disease type and severity, to assess its diagnostic significance and to provide further evidence regarding the underlying pathogenetic process.

## Material and methods

### Patients and tissue samples

Eighty-seven (87) needle core liver biopsies (each longer than 1.5 cm long and containing more than 12 portal tracts) and 5 surgically excised liver specimens from 92 adult patients (57 males (61%), 35 females (39%); median age 54 years, interquartile range 27) were studied. Inclusion criteria were presence of cholestasis defined by histological and/or biochemical findings based on available data on serum levels of liver enzymes (ALT, AST, ALP, GGT) at the time of liver biopsy/surgery. Exclusion criteria were established cirrhosis and liver failure, since possible synergistic factors, such as ischemia, may contribute to aberrant K7 hepatocyte expression. Patients mean international normalised ratio (INR) was 1.15 ± 0.34 (min=0.87-max=2.81).

Tissue samples were retrieved from the archives of the 1^st^ Department of Pathology and the Laboratory of Histology & Embryology, Medical School, National and Kapodistrian University of Athens (NKUoA) between 2012 and 2018. Demographic and biochemical data were retrieved from patient files. All cases were anonymized and permission for scientific use of patient data were obtained by the Research Ethics and Deontology Committee, NKUoA.

Specimens were fixed in 10% neutral formalin solution and processed according to routine protocol. Three-4 μm-thick sections were stained with Hematoxylin & Eosin, Masson trichrome and van Gieson stains. K7 expression was identified immunohistochemically (anti-keratin 7 antibody clone OV-TL12/30, dilution 1:200, Biogenex). Immunostaining for hepatocyte marker HepPar1 (clone OCH1E5, dilution 1:300, DAKO) and cell proliferation marker Ki67 (MIB-1 clone, dilution 1:200, DAKO) were also performed on serial sections, where tissue was available. Double immunostaining for K7 and Ki67 using 3,3-diaminobenzidine (DAB) or 3-amino-9-ethylcarbazole (AEC) chromogens, respectively, was performed in 5 cases of acute hepatitis and 5 cases of pure/mixed cholestasis with available tissue. K19 expression was not examined as it is reportedly absent from hepatocytes in cholestatic liver disease [2, 10].

### Histological and immunohistochemical evaluation

Central histopathology review was performed by two expert liver pathologists (DT, JD). Each case was classified into two major disease groups based on the cause of cholestasis: group I (*n*=36) with parenchymal lesions and group II (*n*=56) with bile duct obstruction/destruction (BDO). Group I was subdivided into two subgroups: acute hepatitis (AH) (*n*= 20, 13 drug-induced and 7 autoimmune), and pure/mixed cholestasis (P/M Chol) (*n*=16, all drug-induced). Group II was subdivided into two subgroups: incomplete bile duct obstruction/destruction (iBDO) including diseases with partial obstruction of bile flow due to primary or secondary cholagiopathy (*n*=48: primary biliary cholangitis-PBC *n*=35, primary sclerosing cholangitis-PSC *n*=10, drug induced vanishing bile duct syndrome-VBDS *n*=3) and complete large bile duct obstruction (cLDBO) due to space-occupying lesions causing complete obstruction to bile flow (*n*=8). Five out of the 8 cLBDO cases were surgical specimens with distal extrahepatic (*n*=3) or perihilar cholangiocarcinoma (*n*=2), while 3 cases were liver biopsies with early LBDO. In the 3 cLBDO biopsy cases, imaging showed dilatation of the right hepatic duct (*n*=2) or the left hepatic duct (*n*=1) that were later ascribed to intraductal papillary neoplasms or periductal infiltrating cholangiocarcinoma, respectively.

Histological features (severity of lobular necroinflammatory activity, portal inflammation, bile duct (BD) loss, bilirubinostasis) were graded semi-quantitatively using a 4-tier scale (grade 0–3). Hepatocellular expression of K7 was assessed in acinar zone 1 and in zones 2 and 3 combined. Hepatocellular Ki67 labelling index (LI) was evaluated in 12/20 AH, 10/16 P/M Chol, 30/48 iBDO and 5/8 cLDBO cases.

Ductular reaction (DR) was evaluated in 82 cases, as previously described [7, 23]. Hepatic progenitor cell (HPC) DR was defined as irregular cords of small cuboidal or elongated cells with or without poorly discernible lumina, located at the portal–parenchyma interface with or without extension to the periphery of hepatic lobules. Interspersed isolated, small K7-positive cells with scant cytoplasm and oval nuclei may be present. DR type 2A according to Desmet [20], proposed to originate from biliary metaplasia of hepatocytes, was also recorded.

Details on semi-quantitative grading for each feature and fibrosis stage are as follows: *K7 immunohistochemical expression scoring*: Zone 1: score 0 absent K7 immunostain in periportal hepatocytes, score 1 ≤10 K7-positive periportal hepatocytes in ≥1 portal tract, score 2 >10 and up to 50% periportal hepatocytes irrespective of number of portal tracts involved or >50% periportal hepatocytes in <50% of portal tracts, score 3 ≥50% of periportal K7-positive hepatocytes around ≥50% of portal tracts; Zone 2 and 3: score 0 absent K7 immunostain in Zone 2 and 3 hepatocytes, score 1 ≤10% K7-positive hepatocytes in at least 1 acinus, score 2 11–30% K7-positive hepatocytes in at least 1 acinus, score 3 >30% K7-positive hepatocytes in at least 1 acinus. Scores ≥2 irrespective of zonal topography corresponded to high hepatocellular K7 expression.

- *Ki67 labelling index (LI):* mean percentage of hepatocytes with nuclear immunostaining/10 high power optical fields (OF) x40
- *Lobular necroinflammatory activity grade*: Grade 0 ≤1 necroinflammatory focus/×10 OF, 1 (minimal/mild) 2–10 necroinflammatory foci/×10 OF, 2 (moderate) >10 necroinflammatory foci/×10 OF, 3 (severe) confluent necrosis.
- *Portal inflammation grade (based on Ishak modified histological activity index)*: Grade 0 (absent), 1 (mild), 2 (moderate), 3 (marked).
- *Fibrosis stage*: Stage 0 no fibrosis, stage 1 portal/periportal fibrosis with or without short fibrous septa, stage 2 portal/periportal fibrosis with few bridging fibrous septa (≤2/1 cm), stage 3 advanced bridging fibrosis (>2 fibrous septa/cm).
- *Bilirubinostasis*: Grade 0 (absent), 1 (mild) poorly discernible at low magnification, grade 2 (moderate) and 3 (marked) easily recognisable at low magnification, grade 3 panacinar extent and high density.
- *BD loss*: Number of portal tracts with no BD to total number of complete portal tracts: Grade 0 no bile duct loss, 1 <1/3, 2 1/3–2/3, 3 >2/3 of portal tracts without a bile duct.
- *Ductular reaction (DR)*: Grade 1 focal, discontinuous or continuous DR in <1/3 of the portal tract circumference, grade 3 continuous DR rimming >1/3 of the circuference in ≥50% of portal tracts. Grade 2 DR was intermediate between grades 1 and 3 [7]

### Statistical analysis

Statistical analysis was performed using the SPSS software (version 19.0, SPSS Inc., Chicago, IL, USA). Data were expressed as frequencies, mean with SD or median with interquartile range (IQR), as appropriate. Quantitative variables were compared with Student’s *t* test or Mann–Whitney test for normally distributed and non-normally distributed variables, respectively. Qualitative variables were compared with the Chi-squared test or Fisher’s exact test, as appropriate. Relationships between parameters were assessed using Spearman’s correlation coefficient. Multivariate logistic regression analysis models were applied to identify independent, significant, predictive factors. All tests were two-sided and *p* values < 0.05 were considered significant.

For statistical analysis, K7-positivity scores 2 and 3 were merged into a single score mentioned in the text as ‘high’ K7 hepatocellular expression.

## Results

**Fig. 1:**
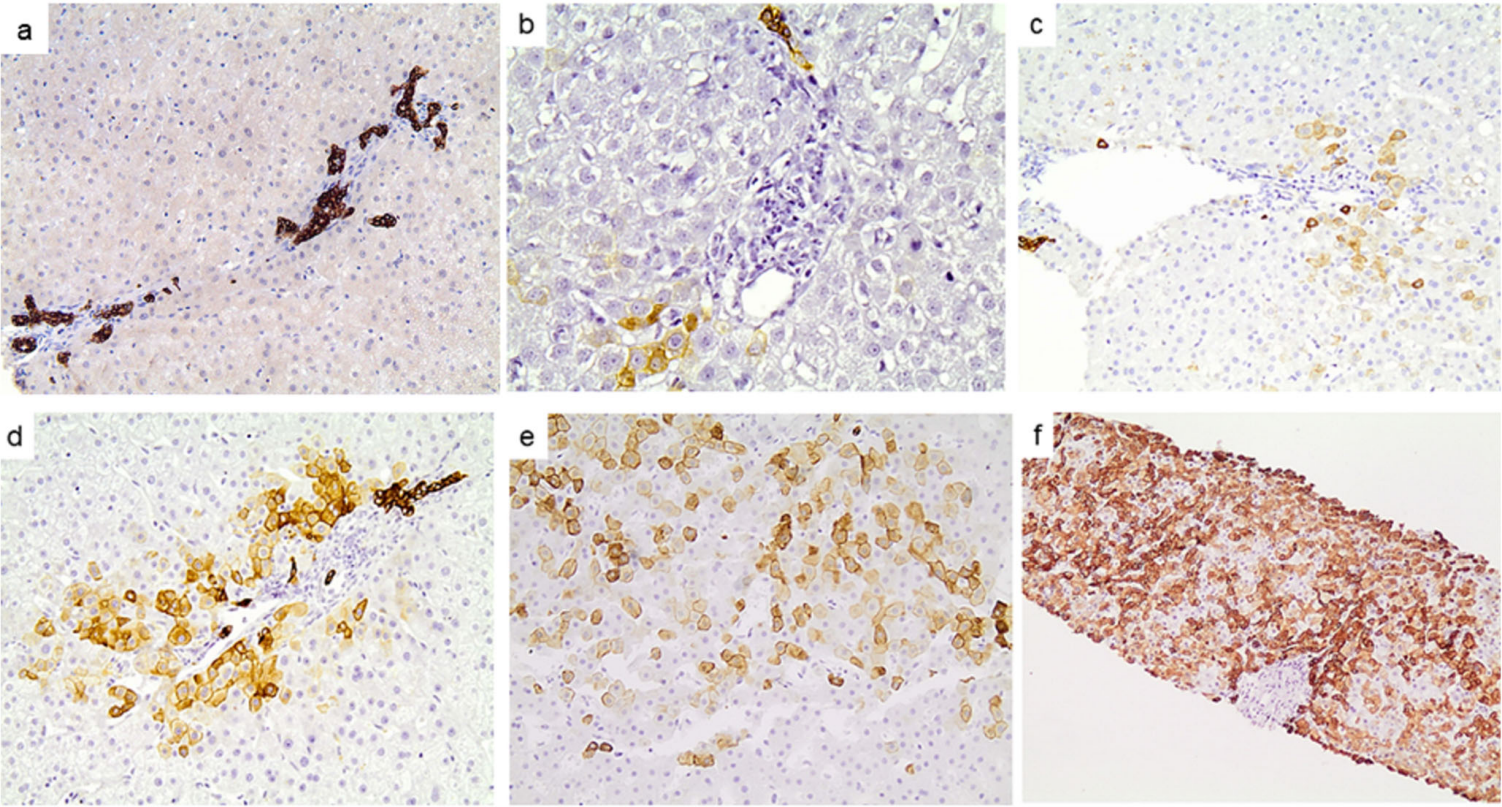
Representative scores of keratin 7 (K7) immunoexpression in cholestatic diseases of different aetiology. **a** Score 0: absence of K7 immunostain in periportal hepatocytes. Bile ducts and ductules are K7-positive; **b** Zone 1, score 1: fewer than 10 K7-positive hepatocytes around a single portal tract in a case of PBC. Note a K7-positive ductule and absence of interlobular bile duct; **c** Zone 1, score 2: more than 10 periportal hepatocytes are K7-positive in one of few similar portal tracts in a case of PSC; **d** Zone 1, score 3: the majority of periportal hepatocytes are K7-positive around a representative portal tract in a case of PSC where ≥50% of portal tracts had a similar appearance; **e** Zones 2 and 3, score 3: >30% of hepatocytes are positive in one representative acinus in a case of drug-induced mixed cholestasis; **f** Zone 1, score 3 and Zones 2 and 3, score 3. Panacinar K7 expression in a case of drug-induced vanishing bile duct syndrome (**a** ×100, **b** ×400, **c**–**e** ×200, **f** ×40 magnification)

The earliest sign of K7 hepatocellular expression was weak cytoplasmic immunoreactivity, which gradually evolved to more intense cytoplasmic and membranous immunostaining occasionally accompanied by diminished cell size. Representative examples of hepatocellular K7 expression scores 0–3 are depicted in Fig. 1.

Hepatocellular K7 positivity was observed in 81/92 (87%) of the examined cases. Variable numbers of K7-positive hepatocytes were noted in 80% AH, 75% P/M Chol, 95.8% iBDO and 87.5% of cLBDO cases. High K7 expression (≥2) irrespective of zonal topography was encountered in 55/92 (60%) cases, including 25% AH, 37.5% of P/M Chol, all PSC cases, 74.3% PBC, 2/3 VBDS and 75% cLBDO cases. Table 1 summarises zonal hepatocellular K7 expression in all diagnostic groups and subgroups.

**Table 1:** Hepatocellular keratin 7 expression in all diagnostic categories and subgroups

	K7 hepatocellular expression					
	All zones		Zone 1		Zones 2 & 3	
	Score ≥1 n (%)	Score ≥2 n (%)	Score ≥1 n (%)	Score ≥2 n (%)	Score ≥1 n (%)	Score ≥2 n (%)
All patients (n=92)	81 (87)	55 (60)	79 (86)	54 (60)	67 (73)	10 (32.6)
Acute hepatitis (n=20)	16 (80)	5 (25)	15 (75)	4 (20)	11 (55)	5 (25)
Pure/mixed cholestasis (n=16)	12 (75)	6 (37.5)	11 (68.8)	6 (37.5)	12 (75)	3 (18)
iBDO (n=48)	46 (96)	38 (79)	46 (96)	38 (79)	38 (79)	17 (35.4)
PBC (n=35)	34 (97.1)	26 (74.35)	34 (97)	26 (74.3)	26 (74.3)	10 (28.6
PSC (n=10)	10 (100)	10 (100)	10 (100)	10 (100)	10 (100)	5 (50)
VBDS (n=3)	2 (66.7)	2 (66.7)	2 (66.7)	2 (66.7)	2 (66.7)	2 (66.7)
cBDO (n=8)	7 (87.5)	6 (75)	7 (87.5)	6 (75)	6 (75)	5 (62.5)

“All zones” scores were calculated using the highest score in each case; *K7* keratin 7, *n* number of cases, *Z* acinar zone, *iBDO* incomplete Bile Duct Obstruction/Destruction, *PBC* Primary Biliary Cholangitis, *PSC* Primary Sclerosing Cholangitis, *VBDS* Vanishing Bile Duct Syndrome, *cBDO* complete large Bile Duct Obstruction

Data on biochemical parameters for each disease type are shown in *Supplementary Table* 1.

### Relationship of Κ7 hepatocellular expression with type of cholestasis, histological parameters, hepatocellular proliferative activity and HepPar1 expression

#### Type of cholestasis

The type of cholestasis (obstructive or parenchymal) correlated significantly with K7-positivity and zone 1 predilection. Cases with bile duct obstruction showed significantly higher K7 hepatocellular expression compared to cases with parenchymal lesions (78.6% vs 30.6%, *p*<0.001). This difference was sustained in the subgroups with K7 expression in zone 1 hepatocytes (94.6% vs 72.2%, *p*=0.004), and was stronger when high K7 expression was considered (78.6% vs 27.8%, *p*<0.001). In the comparative analysis of K7 expression in zones 2–3, a numerical difference was found that did not reach statistical significance.

#### Bile duct loss

Hepatocellular K7 expression in relation to BD loss is summarised in Table 2. BD loss correlated significantly with the presence of K7-positive hepatocytes (97.3% with vs 82% without BD loss, *p=*0.045). This correlation was stronger in the subgroup of cases with high K7 expression (86.5 vs 42%, *p*<0.001). Zone 1 K7-positivity was more common in cases with BD loss compared to the group without (97.3% vs 78%, *p*=0.013). K7 expression in zones 2–3 exhibited nearly significant correlation with marked BD loss (100% with moderate/high vs 82% with low/absent BD loss, *p=*0.064).

**Table 2:** Hepatocellular keratin 7 expression in correlation to bile duct loss and fibrosis stage

		K7 expression					
		Zones 1–3		Zone 1		Zones 2–3	
		Score ≥1 *n* (%)	Score ≥2 *n* (%)	Score ≥1 *n* (%)	Score ≥2 *n* (%)	Score ≥1 *n* (%)	Score ≥2 *n* (%)
BD loss	Grade 0 *n*=55	45 (82)	23 (42)	43 (78)	22 (40)	37 (67.3)	14 (25.5)
	Grade 1–3 *n*=37	36 (97.3)	32 (86.5)	36 (97.3)	32 (86.5)	30 (81)	16 (43)
	Grade 2and 3 *n*=20	20 (100)	18 (90)	20 (100)	18 (90)	20 (100)	10 (50)
Fibrosis stage (F0–3)	F0 *n*=17	14 (82.4)	5 (29.4)	13 (76.5)	5 (29.4)	11 (64.7)	4 (23.5)
	F1-3 *n*=75	67 (89.3)	50 (66.7)	66 (88)	49 (65.3)	56 (74.7)	26 (34.7)
	F0-2 *n*=*71*	60 (84.5)	37 (52)	58 (81.7)	36 (50.7)	48 (67.6)	18 (25.4)
	F3 *n*=21	21 (100)	18 (85.7)	21 (100)	18 (85.7)	19 (90.5)	12 (57)

*K7* keratin 7, *BD* bile duct, *n* number of cases

#### Fibrosis stage

Hepatocellular K7 expression in relation to fibrosis stage is summarised in Table 2. Patients with any liver fibrosis exhibited more often high K7 expression irrespective of zonal topography compared to those without fibrosis (66.7% vs 29.4%, *p=*0.006). All cases (100%) with advanced fibrosis (F3) showed hepatocellular K7 positivity, very often high (85.7%) compared to lower frequency and positivity in lower stages (F0-2) (84.5%, *p*=0.048, and 52%, *p*=0.006 respectively). In advanced fibrosis, zone 1 K7 positivity was more frequent compared to lower stages (100% vs 81.7%, *p*=0.035), irrespective of underlying cholestatic disease aetiology. In zone 1, this difference was stronger when K7 expression was high (85.7% vs 50.7%, *p*=0.005). In zones 2–3, detectable K7 positivity was more often encountered in advanced fibrosis compared to lower fibrosis stages (90.5 % vs 67.6%, *p*=0.050) and this difference was stronger for high K7 positivity (57% vs 25.4%, *p*=0.009).

#### Hepatocellular proliferative activity and HepPar1 expression

HepPar1 expression was well preserved in all hepatocytes of the acini including K7-positive hepatocytes as deducted by the presence of diffuse cytoplasmic granular positivity, irrespective of zonal topography and disease group (Supplementary Figs. 1a & 1b). Ηepatocellular Ki67 LI was low (0–5%) in all disease groups except AH (mean 19%, range 3–40%) (Supplementary Figs. 1c & 1d).

#### Multivariate statistical analysis

In multivariate analysis, K7 hepatocellular expression in zone 1 correlated significantly with type of cholestasis (*p*=0.001), fibrosis stage (*p*=0.022) and BD loss (*p*=0.015). A trend was observed between cholestasis type and high K7 expression irrespective of zonal topography (*p*=0.054). No correlation was seen with age or severity of lobular necroinflammation, portal inflammation and bilirubinostasis. K7 expression in zones 2–3 hepatocytes showed no significant correlation with cholestasis type or any of the examined parameters.

High K7 expression in hepatocytes showed a positive correlation with GGT levels irrespective of topography (*R*=0.283, *p*=0.040), which was stronger for zone 1 (*R*=0.273, *p=*0.035), while ALT levels tended to be inversely correlated (*R*=−0.236, *p*=0.088). Multivariate analysis of K7 expression in relation to biochemical parameters is shown in Table 3.

**Table 3:** Multivariate analysis comparing keratin 7 hepatocellular expression with biochemical parameters

K7 expression score		All disease groups		Acute hepatitis		Pure-mixed cholestasis		Incomplete bile duct obstruction	
		≥1 *p*	≥2 *p*	≥1 *p*	≥2 *p*	≥1 *p*	≥2 *p*	≥1 *p*	≥2 *p*
Zones 1-3	ALT	0.438	**0.088**	n/a	0.280	0.404	**0.004**	0.105	0.406
	AST	0.536	0.145	n/a	0.237	0.361	0.934	0.311	0.298
	GGT	0.331	**0.040**	n/a	0.823	0.430	0.919	**0.061**	0.593
	ALP	0.788	0.704	n/a	0.800	0.840	0.579	0.106	0.707
	Tot bil	0.670	0.120	n/a	0.997	0.171	0.979	0.416	0.801
Zone 1	ALT	**0.095**	0.102	**0.010**	0.166	0.404	**0.004**	0.406	0.105
	AST	0.152	**0.051**	**0.077**	0.183	0.361	0.934	0.298	0.311
	GGT	0.876	**0.035**	0.456	0.891	0.840	0.570	0.702	0.106
	ALP	0.369	0.807	0.860	0.969	0.430	0.919	0.593	**0.061**
	Tot bil	0.485	0.146	0.943	0.390	0.171	0.976	0.801	0.406
Zones 2–3	ALT	0.156	0.447	0.900	0.165	0.404	0.480	0.214	0.432
	AST	0.257	0.295	0.625	0.164	0.361	0.446	0.624	0.952
	GGT	0.241	0.241	0.957	0.611	0.840	0.756	**0.067**	**0.018**
	ALP	0.349	0.349	0.440	0.713	0.136	0.935	0.100	0.305
	Tot bil	0.119	0.119	0.928	0.147	0.171	0.999	0.514	0.135

*K7* keratin 7, *Tot bil* total bilirubin, *n/a* not applicable

Significant or nearly significant *p* in bold

### Κ7 hepatocellular expression according to cholestatic disease type

#### K7 expression in parenchymal cholestasis

*Supplementary Table* 2 shows detailed results of K7 expression in liver diseases with parenchymal cholestasis according to the studied histological parameters. Statistically significant correlations and trends are shown below according to disease subgroup.

i. *Acute hepatitis*

In acute hepatitis, all 5 cases with moderate necroinflammation expressed K7 while in the more severe 15 cases, K7 was detectable in 66.6% *p*=0.265. High K7 expression in zone 1 was more common in cases with milder lobular necroinflammation (*p*=0.032).

ALT levels were significantly higher in patients without K7 expression [mean values: 2155±387U K7(−) vs 460±428U K7(+), *p*=0.006], while a similar trend was observed for AST levels [mean values: 1943±522U K7(−) vs 426±640U K7(+), *p*=0.055].

**Fig. 2:**
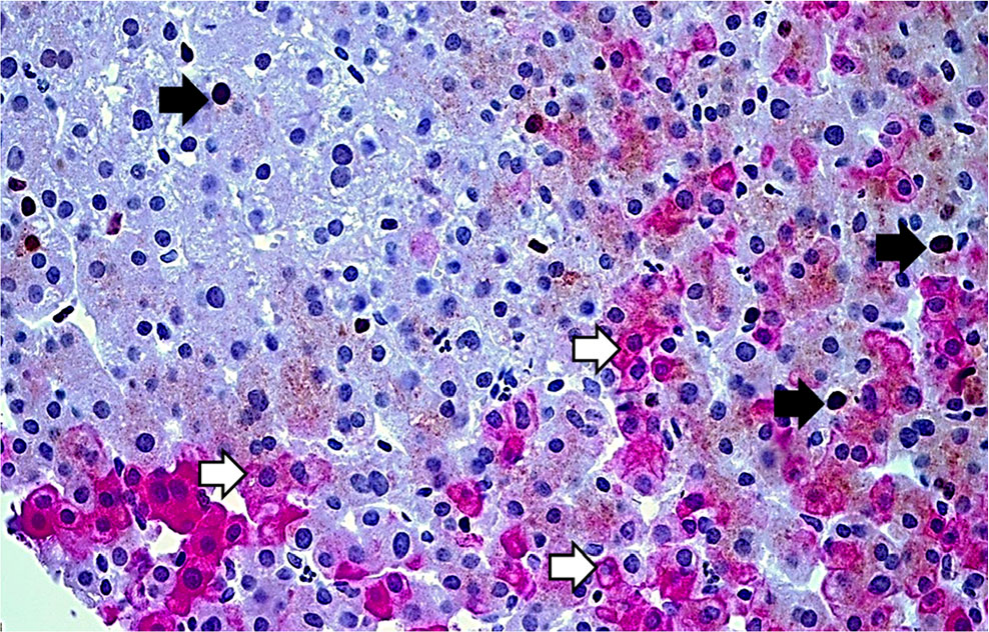
Mild acute hepatitis. Ki67 nuclear immunostaining in keratin 7 (K7)-negative hepatocytes (black arrows) and few non-parenchymal cells. K7-positive hepatocytes (white arrows) are negative. Double Ki67/K7 immunostaining, dark brown DAB (Ki67) and magenda AEC (K7) chromogen, ×400 magnification

In multivariate analysis, K7 expression in zone 1 hepatocytes correlated inversely with the severity of necroinflammation (*p*=0.027). Moreover, transaminase levels were lower in cases with zone 1 K7 expression (ΑST *R=* −0.576, *p*=0.077, ALT *R=*−0.499, *p=*0.010) (Table 3). An inverse correlation was noted between Ki67 proliferation index and K7 score (*R*=−0.609, *p=*0.035). In Ki67/K7 double immunostained cases, Ki67 was nearly absent in K7-positive hepatocytes and was mainly detectable in K7-negative hepatocytes or non-parenchymal cells (Fig. 2).

ii. *Pure/mixed cholestasis*

Cases with moderate or severe bilirubinostasis showed more often K7 positivity as well as high K7 expression in zone 1 compared to cases with mild (90.9% vs 40%, *p*=0.063, 62.5% vs 12.5%, *p*=0.119 respectively). There was no significant correlation between K7 expression and any of the examined parameters.

In multivariate analysis, a correlation trend was found between detectable K7 in zone 1 and zones 2–3 with fibrosis (*p*=0.074 and *p*=0.069, respectively), as well as a significant inverse correlation between high K7 positivity with ALT levels (*p*=0.004) (Table 3).

#### K7 expression in obstructive cholestasis

*Supplementary Table* 3 shows detailed results of K7 expression in liver diseases with obstructive cholestasis according to the studied histological parameters. Statistically significant correlations and trends are shown below according to disease subgroup.

i. *Incomplete bile duct obstruction*

In iBDO, K7-positive hepatocytes were almost constantly present in zone 1 (95.8%), while in zones 2–3, their presence decreased to 78% of the cases (*p*=0.040). Patients showing any percentage of BD loss compared to patients without BD loss tended to exhibit more frequently high K7 positivity in zone 1 (86% vs 58.3%, *p*=0.096). High K7 expression in zones 2–3 was also found more often in patients with BD loss (41.7% vs 16.7%, *p*=0.169) and in advanced fibrosis (F3) (50% vs 26.6%, *p*=0.127).

In multivariate analysis, only age and GGT levels correlated with K7 expression. In detail, patients with high K7 expression in zone 1 and/or zones 2–3 where younger than patients with low K7 positivity (48.7±15.4 years vs 60.3±8.9 years, *p*=0.066). The correlation was stronger in high K7 expression in zone1 or zones 2–3 (Z1 60.3+8.9 vs 48.7+15.4, *p*=0.039. Z 2–3 48.2±14.7 years vs 60.6±12 years, *p*=0.037). Moreover, high K7 expression, irrespective of topography, showed nearly significant correlation with GGT levels (*R*= 0.344, *p*=0.061).

ii. *PBC vs PSC*

All PSC cases and 97.1% PBC cases showed K7-positive hepatocytes. Moreover, all PSC patients showed high K7 expression in zone 1 and/or zones 2–3 compared to PBC (74.3%), *p*=0.173. In cases without BD loss, PSC cases had more frequently high hepatocellular K7 expression compared to PBC (100% vs 20%, *p*=0.015). No difference was noted between PSC and PBC cases regarding high K7 positivity in advanced fibrosis. There were no differences in K7 expression in the different zones.

iii. *Complete bile duct obstruction*

Almost all complete LBDO cases (7/8) showed K7 hepatocellular positivity that was high in the majority (6/8). No further statistical analysis was performed due to the small sample size.

#### K7 hepatocellular expression and ductular reaction

Supplementary Tables 4 and 5 summarise the type and grade of DR observed in parenchymal and obstructive cholestatic diseases, respectively. Briefly, in acute hepatitis, 26% (5/19) of the cases had no DR while DR type 2A was only seen in 1/19 case. No correlation was found with K7 positivity in zone 1 even in cases with high K7 expression (*p*=0.225).

In pure/mixed cholestasis, DR type 2A was noted in 5/15 cases, all of which had high K7 zone 1 expression, while low K7 positivity (9/10) was seen in cases without DR type 2A (*p*=0.002).

In PBC, the vast majority of cases had DR (Supplementary Fig. 2). In the PBC cases with DR type 2A (11/31), there was statistically significant high K7 expression in zone 1 (11/11, 100% vs 12/20, 60%, *p*=0.028).

In PSC, 6/9 cases (66%) showed DR type 2A, while in 3 cases with ductopenia and high K7 zone 1 expression, there was no DR. The latter was observed in 1 out of the two VBDS cases while the second showed grade 3 DR type 2A with high K7 zone 1 hepatocyte expression.

In cLBDO, DR type 2A was seen in all cases and was grade 3 in the majority (4/6) with high K7 zone 1 hepatocyte expression (Supplementary Fig. 3). Two cases with early obstructive changes had grade 1 DR type 2A associated with low K7 zone 1 hepatocyte expression.

In acute hepatitis, pure/mixed cholestasis and PBC*,* K7-positive zone 1 hepatocytes were often found in areas deprived of DR.

## Discussion

The findings of our study complemented by those in the literature suggest that K7 expression in hepatocytes is a common phenomenon in acute and chronic liver diseases. In chronic diseases, such as chronic viral hepatitis, autoimmune hepatitis and steatohepatitis, this cellular alteration is limited [1, 2, 7, 10] while in cholestasis and in ischemia may be widespread. A zone 3 predominant distribution pattern has been described in venous stasis, while non-alcoholic and alcoholic steatohepatitis in association with centrilobular scarring is accompanied by K7-positive centrilobular hepatocytes [1, 6, 16]. In autoimmune and chronic viral hepatitis, K7-positive hepatocytes are scattered in the lobules and their number increases in parallel with the number of HPC, fibrosis stage and disease activity [6, 8]. In chronic cholestasis, K7 hepatocellular expression increases with disease progression and is extensive in cases with longer duration [2, 13, 21]

In previous studies, hepatocellular K7 expression has been investigated in the context of PBC or various non-cholestatic diseases, while only a small series included cases with cholestasis of different aetiology [13]. Assessment and comparative analysis of K7 expression in parenchymal and obstructive cholestasis regarding extension, distribution pattern, other histological factors and serum biochemistry have not been addressed to date.

In our study, K7 hepatocellular expression was seen in all types of cholestatic diseases with the lowest incidence in pure/mixed cholestasis and the highest in iBDO, reaching 100% in PSC. The preferential topography was zone 1 (86%), while zones 2–3 followed (73%) and had less frequent high K7 expression. Cholestasis type and BD loss were independent factors associated with K7 expression. Cases with obstructive cholestasis showed higher K7 positivity and predominance of zone 1 topography compared to those with parenchymal cholestasis. Almost all cases with BD loss expressed K7 showing significant higher frequency and higher positivity compared to those with intact BD. Zone1 K7 topography was almost constantly present in patients with BD loss while zones 2 and 3 were involved in all cases with significant BD loss. Advanced fibrosis also emerged as an independent parameter correlating with K7 hepatocellular expression, mainly of high positivity in zone 1. A similar correlation was found regarding K7 expression in zones 2 and 3, though in lower frequency. HepPar1 expression was not affected, irrespective of cholestatic disease and extent of K7 hepatocellular positivity.

Acinar zone 1 appears to be most vulnerable under normal and cholestatic conditions due to the highest bile salt level exposure. Bile salt cumulative concentration most likely accounts for the high frequency and high zonal expression of K7, while bilirubinostasis and serum bilirubin levels did not exhibit any relationship with K7 hepatocellular expression in our analysis. The differences in the distribution pattern and extent of K7 expression between our study group and non-cholestatic diseases, based on the results of respective studies, suggest different pathogenetic mechanisms.

Analysing each of the examined disease entities, there are certain findings to be highlighted. In acute hepatitis, an inverse correlation of K7-positive hepatocytes with histological disease severity was corroborated by an inverse correlation with serum transaminase levels. In a clinico-pathological study of autoimmune hepatitis (AIH) with acute clinical onset by Fujiwara et al., a higher number of intermediate hepatocytes and HPC was found in non-severe recovered cases compared to severe or fulminant AIH with fatal outcome [12]. It was suggested that this difference reflected efficient regeneration through differentiation of periportal HPCs to intermediate cells and mature hepatocytes. The results of double immunostaining for K7 and Ki-67 antigen in our study showed that K7-positive hepatocytes are characterised by low replicative state, supporting the view that they may predominantly rise through metaplasia, as discussed in detail below. This difference could be attributed to the fact that patient groups in the two studies are not fully comparable, as Fujiwara et al. included clinically severe and fulminant hepatitis cases. Therefore, despite similar histological findings, different pathogenetic backgrounds may underlie the emergence of K7-positive hepatocytes in the two studies.

In drug-induced pure/mixed cholestasis, multivariate analysis showed a correlation trend between fibrosis and detectable hepatocellular K7 expression in all zones, possibly indicating a long-term impact of cholestasis on the manifestation of K7. This is supported by a previous study of 10 cases with pure cholestasis, half of them with predominant zone 1 K7-positivity and histology favouring long duration of the cholestatic lesions [13]. The inverse correlation of K7 expression with ALT levels may have a similar explanation to that presented above for acute hepatitis.

K7 hepatocellular expression has been best studied in incomplete BDO and mainly in PBC while information referring to PSC is limited [14, 17, 22, 24] In PBC, K7 expression has been shown to increase with disease progression [2] and to correlate with Ludwig disease stages [10, 13, 21]. In early stages, it appears restricted in zone 1, while it is more widespread in advanced disease [2]. We showed a very high frequency of K7-positivity in iBDO cases, not only in zone 1 where it was almost constantly encountered but also in zones 2–3. High K7 expression in zone 1 and zones 2–3 was associated with BD loss of any degree and/or advanced fibrosis. Younger patient age was an independent factor of high K7 hepatocellular expression, while GGT levels showed a nearly significant correlation. Comparison between K7 positivity and biliary enzymes is mentioned in one study showing poor correlation [21].

Our findings highlight differences between PBC and PSC regarding K7 expressing hepatocytes. All patients with PSC and almost all with PBC showed K7-positive hepatocytes. It is noteworthy that all PSC cases including those without bile duct loss exhibited high K7 hepatocyte expression compared to only 20% of PBC cases with intact bile ducts, a feature that may prove helpful in the differential diagnosis of cholestatic syndromes without apparent BD loss in liver biopsy.

Regarding the nature of K7-positive hepatocytes, based on Ki67 proliferation activity, they seem to be resting cells, retaining hepatocellular features, such as HepPar1 expression, as well as functional properties. The latter is deducted by the absence of clinical and biochemical findings of liver insufficiency in cases with almost panacinar K7 expression. According to our observations in parenchymal cholestasis, K7-positive hepatocytes do not inevitably undergo biliary transformation. The latter process, characterised primarily by ductule formation in zone 1, involves the deeper zones of the acinus especially in advanced stages of BDO (data not shown). In all cholestatic diseases, including parenchymal lesions, the vast majority of K7-positive hepatocytes most likely result from a metaplastic process and not from hepatocellular differentiation of HPC. This is supported by their strong association with DR type 2A, their presence in the cases without DR and their frequent periportal topography independent of DR location. Most likely, K7 expression in hepatocytes reflects a protective mechanism attributed to the established cytoprotective role of intermediate filaments [25]. It may be triggered by cytokines and xenobiotics in inflammatory and bland cholestasis and by the toxic effect of bile constituents, especially bile salts, in all types of cholestasis [26, 27]. The fact that younger age correlated with extensive K7-positivity indicates a diminished adjustment capacity of aging hepatocytes.

In summary, K7 hepatocellular expression in cholestatic diseases is a sensitive though not specific marker of cholestasis related to type of cholestasis, fibrosis and BD loss. Our study shows that it may represent a cytoprotective reaction of resting hepatocytes in cholestasis of longer duration without elimination of functional and other phenotypic features, especially in younger patients.

## Supplementary Information

ESM 1 (DOCX 20.4 kb)

ESM 2 (DOCX 36.5 kb)

ESM 3 (DOCX 46.8 kb)

ESM 4 (DOCX 22.8 kb)

ESM 5 (DOC 67.0 kb)

Supplementary Figure 1 a & c. The majority of hepatocytes around representative portal tracts are Keratin 7 (K7)-positive in a case of PBC where ≥50% of portal tracts had a similar appearance (Score 3); b. Serial section of 1a immunostained for HepPar1. All hepatocytes, including those expressing K7, show diffuse cytoplasmic granular HepPar1 immunostaining; d. Serial section of 1c immunostained for Ki-67. K7-positive hepatocytes are negative for Ki-67 indicating that they are resting cells (a, b x100, c, d x200 magnification). (PNG 8664 kb)

High resolution image (TIF 1917 kb)

Supplementary Figure 2 Primary biliary cholangitis: Ductular reaction grade 3 and absence of K7-positive zone 1 hepatocyte expression (x100 magnification). (PNG 6737 kb)

High resolution image (TIF 1546 kb)

Supplementary Figure 3 Complete large bile duct obstruction: ductular reaction type 2A with score 3 K7-positive hepatocyte expression (x400 magnification). (PNG 6701 kb)

High resolution image (TIF 1625 kb)

## Data Availability

All research data and material will be made available upon request. Most data are included in the main manuscript and supporting files.

## References

[1] Matsukuma S, Takeo H, Utsumi Y, Sato K (2017). In hepatic venous outflow obstruction, alcoholic liver disease, and nonalcoholic fatty liver disease, centrilobular scars, CD34+ vessels, and keratin 7+ hepatocytes are in close proximity. Virchows Arch.

[2] Bateman AC, Hübscher SG (2010). Cytokeratin expression as an aid to diagnosis in medical liver biopsies. Histopathology.

[3] Michalopoulos GK (2018). The regenerative altruism of hepatocytes and cholangiocytes. Cell Stem Cell.

[4] Schaub JR, Huppert KA, Kurial SNT, Hsu BY, Cast AE, Donnelly B, Karns RA, Chen F, Rezvani M, Luu HY, Mattis AN, Rougemont AL, Rosenthal P, Huppert SS, Willenbring H (2018). De novo formation of the biliary system by TGFβ-mediated hepatocyte transdifferentiation. Nature.

[5] Chen J, Chen L, Zern MA, Theise ND, Diehl AM, Liu P, Duan Y (2017). The diversity and plasticity of adult hepatic progenitor cells and their niche. Liver Int.

[6] Matsukuma S, Takeo H, Kono T, Nagata Y, Sato K (2012). Aberrant cytokeratin 7 expression of centrilobular hepatocytes: a clinicopathological study. Histopathology.

[7] Delladetsima J, Alexandrou P, Giaslakiotis K, Psichogiou M, Hatzis G, Sypsa V, Tiniakos D (2010). Hepatic progenitor cells in chronic hepatitis C: a phenomenon of older age and advanced liver disease. Virchows Arch.

[8] Eleazar JA, Memeo L, Jhang JS, Mansukhani MM, Chin S, Park SM, Lefkowitch JH, Bhagat G (2004). Progenitor cell expansion: an important source of hepatocyte regeneration in chronic hepatitis. J Hepatol.

[9] Libbrecht L, Desmet V, Van Damme B (2000). Deep intralobular extension of human hepatic “progenitor cells” correlates with parenchymal inflammation in chronic viral hepatitis: can “progenitor cells” migrate?. J Pathol.

[10] Goldstein NS, Soman A, Gordon SC (2001). Portal tract eosinophils and hepatocyte cytokeratin 7 immunoreactivity helps distinguish early-stage, mildly active primary biliary cirrhosis and autoimmune hepatitis. Am J Clin Pathol.

[11] Delladetsima JK, Kyriakou V, Vafiadis I, Karakitsos P, Smyrnoff T, Tassopoulos NC (1995). Ductular structures in acute hepatitis with panacinar necrosis. J Pathol.

[12] Fujiwara K, Nakano M, Yasui S, Okitsu K, Yonemitsu Y, Yokosuka O (2011). Advanced histology and impaired liver regeneration are associated with disease severity in acute-onset autoimmune hepatitis. Histopathology.

[13] Van Eyken P, Sciot R, Desmet VJ (1989). A cytokeratin immunohistochemical study of cholestatic liver disease: evidence that hepatocytes can express “bile duct-type” cytokeratins. Histopathology.

[14] Quaglia A, Bhathal PS (2017). Copper, copper-binding protein and cytokeratin 7 in biliary disorders. Histopathology.

[15] Delladetsima I, Sakellariou S, Kokkori A, Tiniakos D (2016). Atrophic hepatocytes express keratin 7 in ischemia-associated liver lesions. Histol Histopathol.

[16] Krings G, Can B, Ferrell L (2014). Aberrant centrizonal features in chronic hepatic venous outflow obstruction. Am J Surg Pathol.

[17] Zen Y, Hübscher SG, Nakanuma Y (2017) Bile duct diseases. In: Burt AD, Ferrell LD, Hubscher SG (eds) MacSween’s Pathology of the liver, 7^th^ ed. Elsevier Ltd, p 515–593

[18] Zollner G, Trauner M (2008). Mechanisms of cholestasis. Clin Liver Dis.

[19] Hirschfield GM, Heathcote EJ, Gershwin ME (2010). Pathogenesis of cholestatic liver disease and therapeutic approaches. Gastroenterology.

[20] Desmet VJ (2011). Ductal plates in hepatic ductular reactions. Hypothesis and implications. I. Types of ductular reaction reconsidered. Virchows Arch.

[21] Yabushita K, Yamamoto K, Ibuki N, Okano N, Matsumura S, Okamoto R, Shimada N, Tsuji T (2001). Aberrant expression of cytokeratin 7 as a histological marker of progression in primary biliary cirrhosis. Liver.

[22] Kasper HU, Drebber U, Dienes HP (2010). Biliary cytokeratin expression but not CD56 (N-CAM) expression aids in the differential diagnosis of non-neoplastic bile duct diseases. Pathol Res Pract.

[23] Gouw ASH, Clouston AC, Theise ND (2011). Ductular reactions in human liver: diversity at the interface. Hepatology.

[24] Lepistö A, Kivistö S, Kivisaari L, Arola J, Järvinen HJ (2009). Primary sclerosing cholangitis: outcome of patients undergoing restorative proctocolecetomy for ulcerative colitis. Int J Color Dis.

[25] Strnad P, Stumptner C, Zatloukal K, Denk H (2008). Intermediate filament cytoskeleton of the liver in health and disease. Histochem Cell Biol.

[26] Arrese M, Ananthanarayanan M (2004). The bile salt export pump: molecular properties, function and regulation. Pflugers Arch - Eur J Physiol.

[27] Trauner M, Wagner M, Fickert P (2005). Molecular regulation of hepatobiliary transport systems: clinical implications for understanding and treating cholestasis. J Clin Gastroenterol.

