# Supplementary material for: Keratin 7 expression in hepatic cholestatic diseases
Source: Virchows Arch. 2021 Jul 27;479(4):815–24. doi: 10.1007/s00428-021-03152-z (PMC8516784; doi:10.1007/s00428-021-03152-z)
Supplement: Supplementary file 1 — (DOCX 20.4 kb) [file 428_2021_3152_MOESM1_ESM.docx]

**Supplementary Table 1**

[**Virchows Archiv**](https://www.springer.com/journal/428/)

**Keratin 7 expression in hepatic cholestatic diseases**

Sakellariou S^1*^, Michaelides C^1*^, Voulgaris T^2^, Vlachogiannakos J^2^, Manesis E^3^, Tiniakos DG^4,5^**, Delladetsima I^1^**

*Joint first authors, **Joint senior authors

1. 1^st^ Department of Pathology, Medical School, Laiko General Hospital, National and Kapodistrian University of Athens, Athens, Greece

2. Academic Department of Gastroenterology and Hepatology, Laiko General Hospital, National and Kapodistrian University of Athens, Athens, Greece

3. Liver Unit, Euroclinic, Athens Greece

4. Department of Pathology, Aretaieion Hospital, National and Kapodistrian University of Athens, Athens, Greece

5. Translational & Clinical Research Institute, Faculty of Medical Sciences, Newcastle University, Newcastle upon Tyne, United Kingdom

Liver enzyme and total bilirubin serum levels (mean+/- SD) according to cholestatic disease type

|  | **All disease groups** | **Acute hepatitis** | **Pure-mixed cholestasis** | **Incomplete bile duct obstruction** | **Complete bile duct obstruction** |
| --- | --- | --- | --- | --- | --- |
| **ALT** (U/L) | 205±361 | 625±660 | 154±139 | 61±48 | 270±222 |
| **AST** (U/L) | 207±449 | 577±770 | 166±145 | 46±25 | 425±718 |
| **GGT**(U/L) | 309±345 | 153±143 | 367±328 | 221±231 | 653±591 |
| **ALP** (U/L) | 299±262 | 242±136 | 592±337 | 277±299 | 384±197 |
| **Total bilirubin** (mg/dl) | 5.5±8.4 | 9.8±9.7 | 13.8±12.4 | 1.3±3.0 | 6.8±5.9 |

*Normal range ALT 7-55 U/L, AST 8-48 U/L, ALP 40-129 U/L, GGT 8-61 U/L, total bilirubin <1.20 mg/dl*
