# Supplementary material for: Keratin 7 expression in hepatic cholestatic diseases
Source: Virchows Arch. 2021 Jul 27;479(4):815–24. doi: 10.1007/s00428-021-03152-z (PMC8516784; doi:10.1007/s00428-021-03152-z)
Supplement: Supplementary file 2 — (DOCX 36.5 kb) [file 428_2021_3152_MOESM2_ESM.docx]

**Supplementary Table 2**

[**Virchows Archiv**](https://www.springer.com/journal/428/)

**Keratin 7 expression in hepatic cholestatic diseases**

Sakellariou S^1*^, Michaelides C^1*^, Voulgaris T^2^, Vlachogiannakos J^2^, Manesis E^3^, Tiniakos DG^4,5^**, Delladetsima I^1^**

*Joint first authors, **Joint senior authors

1. 1^st^ Department of Pathology, Medical School, Laiko General Hospital, National and Kapodistrian University of Athens, Athens, Greece

2. Academic Department of Gastroenterology and Hepatology, Laiko General Hospital, National and Kapodistrian University of Athens, Athens, Greece

3. Liver Unit, Euroclinic, Athens Greece

4. Department of Pathology, Aretaieion Hospital, National and Kapodistrian University of Athens, Athens, Greece

5. Translational & Clinical Research Institute, Faculty of Medical Sciences, Newcastle University, Newcastle upon Tyne, United Kingdom

Hepatocellular keratin 7 expression in relation to the severity of histological features in diseases with parenchymal cholestasis.

| **PARENCHYMAL CHOLESTASIS** | | | | | | | | | | | | | | | | | | | | | | |
| --- | --- | --- | --- | --- | --- | --- | --- | --- | --- | --- | --- | --- | --- | --- | --- | --- | --- | --- | --- | --- | --- | --- |
|  | | **LOBULAR NECROINFAMMATION** | | | | **PORTAL INFLAMMATION** | | | | **FIBROSIS STAGE** | | | | **BILIRUBINOSTASIS** | | | | **BILE DUCT LOSS** | | | | |
| **DISEASE** | n | n | G | K7 Z1 | K7 Z2-3 | n | G | K7 Z1 | K7 Z2-3 | n | F | K7 Z1 | K7 Z2-3 | n | G | K7 Z1 | K7 Z2-3 | n | | G | K7 Z1 | K7 Z2-3 |
| **Acute hepatitis** | 20 | 5 | 2 | 2 S1 | 2 S1 | 1 | 0 | 1 S2 | 1 S3 | 11 | 0 | 3 S0 | 4 S0 | 1 | 0 | 1 S0 | 1 S0 | 19 | | 0 | 5 S0 | 9 S0 |
|  |  |  |  | 3 S2 | 2 S2 |  |  |  | |  |  | 5 S1 | 4 S1 |  |  |  | |  |  |  | 11 S1 | 6 S1 |
|  |  |  |  |  | 1 S3 |  |  |  |  |  |  | 3 S2 | 2 S2 |  |  |  |  |  |  |  | 3 S2 | 3 S2 |
|  |  |  |  |  | |  |  |  |  |  |  |  | 1 S3 |  |  |  |  |  |  |  |  | 1 S3 |
|  |  | 15 | 3 | 5 S0 | 9 S0 | 3 | 1 | 2 S1 | 1 S0 | 1 | 1 | 1 S0 | 1 S0 | 5 | 1 | 2 S0 | 2 S0 | 1 | 1 | | 1 S3 | 1 S2 |
|  |  |  |  | 9 S1 | 4 S1 |  |  | 1 S2 | 1 S1 |  |  |  | |  |  | 3 S1 | 2 S1 |  |  |  |  | |
|  |  |  |  | 1 S3 | 2 S2 |  |  |  | 1 S2 |  |  |  |  |  |  |  | 1 S2 |  |  |  |  |  |
|  |  |  | | | | 5 | 2 | 1 S0 | 2 S0 | 8 | 2 | 2 S0 | 5 S0 | 8 | 2 | 3 S0 | 4 S0 |  | | | | |
|  |  |  |  |  |  |  |  | 4 S1 | 3 S1 |  |  | 5 S1 | 1 S1 |  |  | 4 S1 | 3 S1 |  |  |  |  |  |
|  |  |  |  |  |  |  |  |  | |  |  | 1 S3 | 2 S2 |  |  | 1 S2 | 1 S2 |  |  |  |  |  |
|  |  |  |  |  |  | 11 | 3 | 5 S0 | 7 S0 |  | | | | 6 | 3 | 3 S1 | 3 S0 |  |  |  |  |  |
|  |  |  |  |  |  |  |  | 4 S1 | 1 S1 |  |  |  |  |  |  | 2 S2 | 2 S2 |  |  |  |  |  |
|  |  |  |  |  |  |  |  | 1 S2 | 3 S2 |  |  |  |  |  |  | 1 S3 | 1 S3 |  |  |  |  |  |
|  |  |  |  |  |  |  |  | 1 S3 |  |  |  |  |  |  |  |  | |  |  |  |  |  |
| **Pure/Mixed Cholestasis** | 16 | 5 | 0 | 3 S0 | 2 S0 | 6 | 1 | 3 S0 | 3 S0 | 4 | 0 | 1 S0 | 1 S0 | 5 | 1 | 3 S0 | 3 S0 | 16 | | 0 | 5 S0 | 4 S0 |
|  |  |  |  | 1 S2 | 1 S1 |  |  | 1 S1 | 1 S1 |  |  | 2 S1 | 3 S1 |  |  | 1 S1 | 1 S1 |  |  |  | 4 S1 | 9 S1 |
|  |  |  |  | 1 S3 | 1 S2 |  |  | 1 S2 | 1 S2 |  |  | 1 S2 |  |  |  | 1 S2 | 1 S2 |  |  |  | 5 S2 | 2 S2 |
|  |  |  |  |  | 1 S3 |  |  | 1 S3 | 1 S3 |  |  |  | |  |  |  | |  |  |  | 2 S3 | 1 S3 |
|  |  | 11 | 1 | 2 S0 | 2 S0 | 8 | 2 | 2 S0 | 1 S0 | 11 | 1 | 3 S0 | 3 S0 | 3 | 2 | 1 S0 | 1 S0 |  | | | | |
|  |  |  |  | 5 S1 | 8 S1 |  |  | 4 S1 | 7 S1 |  |  | 3 S1 | 5 S1 |  |  | 2 S1 | 2 S1 |  |  |  |  |  |
|  |  |  |  | 3 S2 | 1 S2 |  |  | 2 S2 |  |  |  | 3 S2 | 2 S2 |  |  |  | |  |  |  |  |  |
|  |  |  |  | 1 S3 |  |  |  |  | |  |  | 2 S3 | 1 S3 |  |  |  |  |  |  |  |  |  |
|  |  |  | | | | 2 | 3 | 1 S2 | 1 S1 | 1 | 2 | 1 S0 | 1 S1 | 8 | 3 | 1 S0 | 6 S1 |  |  |  |  |  |
|  |  |  |  |  |  |  |  | 1 S3 | 1 S2 |  |  |  | |  |  | 2 S1 | 1 S2 |  |  |  |  |  |
|  |  |  |  |  |  |  |  |  | |  |  |  |  |  |  | 3 S2 | 1 S3 |  |  |  |  |  |
|  |  |  |  |  |  |  |  |  |  |  |  |  |  |  |  | 2 S3 |  |  |  |  |  |  |

n Number of cases, G Grade, F Fibrosis stage, Z Zone, K7 Keratin 7, K7 Z1 K7 positive hepatocytes in Zone 1, K7 Z2-3 K7 positive hepatocytes in Zones 2&3, S0-S3 K7 hepatocellular expression score
