# Supplementary material for: Keratin 7 expression in hepatic cholestatic diseases
Source: Virchows Arch. 2021 Jul 27;479(4):815–24. doi: 10.1007/s00428-021-03152-z (PMC8516784; doi:10.1007/s00428-021-03152-z)
Supplement: Supplementary file 3 — (DOCX 46.8 kb) [file 428_2021_3152_MOESM3_ESM.docx]

**Supplementary Table 3**

[**Virchows Archiv**](https://www.springer.com/journal/428/)

**Keratin 7 expression in hepatic cholestatic diseases**

Sakellariou S^1*^, Michaelides C^1*^, Voulgaris T^2^, Vlachogiannakos J^2^, Manesis E^3^, Tiniakos DG^4,5^**, Delladetsima I^1^**

*Joint first authors, **Joint senior authors

1. 1^st^ Department of Pathology, Medical School, Laiko General Hospital, National and Kapodistrian University of Athens, Athens, Greece

2. Academic Department of Gastroenterology and Hepatology, Laiko General Hospital, National and Kapodistrian University of Athens, Athens, Greece

3. Liver Unit, Euroclinic, Athens Greece

4. Department of Pathology, Aretaieion Hospital, National and Kapodistrian University of Athens, Athens, Greece

5. Translational & Clinical Research Institute, Faculty of Medical Sciences, Newcastle University, Newcastle upon Tyne, United Kingdom

Grade of hepatocellular keratin 7 expression in relation to histological parameters in diseases with obstructive cholestasis

| **OBSTRUCTIVE CHOLESTASIS** | | | | | | | | | | | | | | | | | | | | | | |
| --- | --- | --- | --- | --- | --- | --- | --- | --- | --- | --- | --- | --- | --- | --- | --- | --- | --- | --- | --- | --- | --- | --- |
|  | | **LOBULAR NECROINFAMMATION** | | | | **PORTAL INFLAMMATION** | | | | **FIBROSIS STAGE** | | | | | **BILIRUBINOSTASIS** | | | | **BILE DUCT LOSS** | | | |
| **DISEASE** | n | n | G | K7 Z1 | K7 Z2-3 | n | G | K7 Z1 | K7 Z2-3 | n | F | K7 Z1 | K7 Z2-3 | | n | G | K7 Z1 | K7 Z2-3 | n | G | K7 Z1 | K7 Z2-3 |
| **Primary biliary cholangitis** | 35 | 2 | 0 | 1 S1 | 2 S1 | 2 | 1 | 1 S2 | 1 S0 | 1 | 0 | 1 S1 | 1 S0 | | 33 | 0 | 1 S0 | 9 S0 | 5 | 0 | 4 S1 | 2 S0 |
|  |  |  |  | 1 S2 |  |  |  | 1 S3 | 1 S3 |  |  |  | | |  |  | 8 S1 | 15 S1 |  |  | 1 S2 | 3 S1 |
|  |  |  |  |  | |  |  |  | |  |  |  |  |  |  |  | 17 S2 | 4 S2 |  |  |  | |
|  |  |  |  |  |  |  |  |  |  |  |  |  |  |  |  |  | 7 S3 | 5 S3 |  |  |  |  |
|  |  | 20 | 1 | 5 S0 | 5 S0 | 13 | 2 | 1 S0 | 4 S0 | 11 | 1 | 2 S1 | 3 S0 | | 2 | 2 | 2 S3 | 1 S1 | 14 | 1 | 1 S0 | 4 S0 |
|  |  |  |  | 9 S1 | 9 S1 |  |  | 4 S1 | 5 S1 |  |  | 8 S2 | 6 S1 | |  |  |  | 1 S3 |  |  | 2 S1 | 7 S1 |
|  |  |  |  | 6 S2 | 1 S2 |  |  | 6 S2 | 3 S2 |  |  | 1 S3 | 2 S2 | |  |  |  |  |  |  | 9 S2 | 2 S2 |
|  |  |  |  |  | 5 S3 |  |  | 2 S3 | 1 S3 |  |  |  |  | |  |  |  |  |  |  | 2 S3 | 1 S3 |
|  |  | 12 | 2 | 1 S0 | 4 S0 | 20 | 3 | 4 S1 | 4 S0 | 13 | 2 | 1 S0 | 3 S0 | |  | | | | 14 | 2 | 2 S1 | 3 S0 |
|  |  |  |  | 2 S1 | 5 S1 |  |  | 10 S2 | 11 S1 |  |  | 2 S1 | 6 S1 | |  |  |  |  |  |  | 7 S2 | 6 S1 |
|  |  |  |  | 7 S2 | 3 S2 |  |  | 6 S3 | 1 S2 |  |  | 6 S2 | 2 S2 | |  |  |  |  |  |  | 5 S3 | 2 S2 |
|  |  |  |  | 2 S3 |  |  |  |  | 4 S3 |  |  | 4 S3 | 2 S3 | |  |  |  |  |  |  |  | 3 S3 |
|  |  | 1 | 3 | 1 S3 | 1 S3 |  | | | | 10 | 3 | 3 S1 | 2 S0 | |  |  |  |  | 2 | 3 | 2 S3 | 2 S3 |
|  |  |  |  |  | |  |  |  |  |  |  | 3 S2 | 4 S1 | |  |  |  |  |  |  |  | |
|  |  |  |  |  |  |  |  |  |  |  |  | 4 S3 | 4 S3 | |  |  |  |  |  |  |  |  |
| **Primary**  **sclerosing cholangitis** | 10 | 2 | 0 | 2 S3 | 2 S3 | 3 | 1 | 2 S2 | 1 S1 | 2 | 1 | 2 S2 | 1 S1 | | 9 | 0 | 5 S2 | 5 S1 | 6 | 0 | 4 S2 | 4 S1 |
|  |  |  |  |  | |  |  | 1 S3 | 1 S2 |  |  |  | 1 S2 | |  |  | 4 S3 | 1 S2 |  |  | 2 S3 | 1 S2 |
|  |  |  |  |  |  |  |  |  | 1 S3 |  |  |  | | |  |  |  | 3 S3 |  |  |  | 1 S3 |
|  |  | 4 | 1 | 3 S2 | 3 S1 | 5 | 2 | 3 S2 | 4 S1 | 1 | 2 | 1 S2 | 1 S1 | | 1 | 1 | 1 S2 | 1 S2 | 2 | 1 | 1 S2 | 1 S2 |
|  |  |  |  | 1 S3 | 1 S2 |  |  | 2 S3 | 1 S3 |  |  |  | | |  |  |  | |  |  | 1 S3 | 1 S3 |
|  |  | 4 | 2 | 3 S2 | 2 S1 | 2 | 3 | 1 S2 | 1 S2 | 7 | 3 | 3 S2 | 3 S1 | |  | | | | 1 | 2 | 1 S2 | 1 S1 |
|  |  |  |  | 1 S3 | 1 S2 |  |  | 1 S3 | 1 S3 |  |  | 4 S3 | 1 S2 | |  |  |  |  |  |  |  | |
|  |  |  |  |  | 1 S3 |  |  |  | |  |  |  | 3 S3 | |  |  |  |  | 1 | 3 | 1 S3 | 1 S3 |
| **Vanishing bile duct syndrome** | 3 | 1 | 0 | 1 S3 | 1 S2 | 3 | 1 | 1 S0 | 1 S0 | 1 | 0 | 1 S3 | 1 S2 | | 1 | 0 | 1 S3 | 1 S2 | 1 | 0 | 1 S1 | 1 S1 |
|  |  | 2 | 1 | 1 S0 | 1 S0 |  |  | 2 S3 | 2 S2 | 1 | 1 | 1 S1 | 1 S1 | | 1 | 1 | 1 S1 | 1 S1 | 2 | 2 | 2 S3 | 2 S2 |
|  |  |  |  | 1 S3 | 1 S2 |  |  |  | | 1 | 3 | 1 S3 | 1 S2 | | 1 | 3 | 1 S3 | 1 S2 |  |  |  | |
| **Complete bile duct obstruction** | 8 | 1 | 0 | 1 S3 | 1 S3 | 1 | 1 | 1 S3 | 1 S2 | 3 | 1 | 1 S1 | 1 S1 | | 4 | 1 | 1 S0 | 2 S0 | 8 | 0 | 1 S0 | 2 S0 |
|  |  |  |  |  | |  |  |  | |  |  | 1 S2 | 1 S2 | |  |  | 1 S1 | 2 S2 |  |  | 1 S1 | 1 S1 |
|  |  |  |  |  |  |  |  |  |  |  |  | 1 S3 | |  |  |  | 2 S3 |  |  |  | 1 S2 | 4 S2 |
|  |  |  |  |  |  |  |  |  |  |  |  |  | | |  |  |  |  |  |  | 5 S3 | 1 S3 |
|  |  | 5 | 1 | 1 S0 | 2 S0 | 3 | 2 | 1 S0 | 1 S0 | 2 | 2 | 1 S0 | 1 S0 | | 2 | 2 | 1 S2 | 1 S1 |  | | | |
|  |  |  |  | 1 S1 | 1 S1 |  |  | 2 S3 | 1 S2 |  |  | 1 S3 | 1 S2 | |  |  | 1 S3 | 1 S2 |  |  |  |  |
|  |  |  |  | 1 S2 | 2 S2 |  |  |  | 1 S3 |  |  |  | | |  |  |  | |  |  |  |  |
|  |  |  |  | 2 S3 |  |  |  |  | |  |  |  |  |  |  |  |  |  |  |  |  |  |
|  |  | 2 | 2 | 2 S3 | 2 S2 | 4 | 3 | 1 S1 | 1 S0 | 3 | 3 | 3 S3 | 2 S2 | | 2 | 3 | 2 S3 | 1 S2 |  |  |  |  |
|  |  |  |  |  | |  |  | 1 S2 | 1 S1 |  |  |  | 1 S3 | |  |  |  | 1 S3 |  |  |  |  |
|  |  |  |  |  |  |  |  | 2 S3 | 2 S2 |  |  |  |  | |  |  |  |  |  |  |  |  |

n Number of cases, G Grade, F Fibrosis stage, Z Zone, Keratin 7 K7, K7 Z1 K7 positive hepatocytes in Zone 1, K7 Z2-3 K7 positive hepatocytes in Zones 2 &3, S0-S3 K7 hepatocellular expression score
