# Supplementary material for: Keratin 7 expression in hepatic cholestatic diseases
Source: Virchows Arch. 2021 Jul 27;479(4):815–24. doi: 10.1007/s00428-021-03152-z (PMC8516784; doi:10.1007/s00428-021-03152-z)
Supplement: Supplementary file 5 — (DOC 67.0 kb) [file 428_2021_3152_MOESM5_ESM.doc]

**Supplementary Table 5**

[**Virchows Archiv**](https://www.springer.com/journal/428/)

**Keratin 7 expression in hepatic cholestatic diseases**

Sakellariou S^1*^, Michaelides C^1*^, Voulgaris T^2^, Vlachogiannakos J^2^, Manesis E^3^, Tiniakos DG^4,5^**, Delladetsima I^1^**

*Joint first authors, **Joint senior authors

1. 1^st^ Department of Pathology, Medical School, Laiko General Hospital, National and Kapodistrian University of Athens, Athens, Greece

2. Academic Department of Gastroenterology and Hepatology, Laiko General Hospital, National and Kapodistrian University of Athens, Athens, Greece

3. Liver Unit, Euroclinic, Athens Greece

4. Department of Pathology, Aretaieion Hospital, National and Kapodistrian University of Athens, Athens, Greece

5. Translational & Clinical Research Institute, Faculty of Medical Sciences, Newcastle University, Newcastle upon Tyne, United Kingdom

Type and grade of ductular reaction (DR) and keratin 7 (K7) zone 1 hepatocyte expression in obstructive cholestatic disease.

| **Cholestatic liver disease**  **Case Number** | **Ductular reaction**  **(HPC)**  **Grade** | **Ductular reaction**  **type 2A**  **Grade** | **Zone 1 K7 hepatocellular expression score** |
| --- | --- | --- | --- |
| **PBC** |  |  |  |
| **1** | 0 | 1 | 2 |
| **2** | 1 | 0 | 1 |
| **3** | 1 | 0 | 2 |
| **4** | 2 | 0 | 1 |
| **5** | 1 | 1 | 3 |
| **6** | 0 | 3 | 3 |
| **7** | 0 | 1 | 3 |
| **8** | 0 | 0 | 1 |
| **9** | 2 | 1 | 2 |
| **10** | 1 | 0 | 2 |
| **11** | 0 | 0 | 2 |
| **12** | 1 | 0 | 0 |
| **13** | 0 | 0 | 2 |
| **14** | 1 | 0 | 2 |
| **15** | 1 | 0 | 2 |
| **16** | 1 | 0 | 2 |
| **17** | 1 | 0 | 2 |
| **18** | 1 | 0 | 2 |
| **19** | 1 | 3 | 2 |
| **20** | 0 | 0 | 3 |
| **21** | 1 | 0 | 1 |
| **22** | 1 | 0 | 1 |
| **23** | 1 | 3 | 2 |
| **24** | 2 | 0 | 2 |
| **25** | 1 | 0 | 3 |
| **26** | 0 | 2 | 3 |
| **27** | 3 | 0 | 1 |
| **28** | 0 | 2 | 3 |
| **29** | 1 | 1 | 2 |
| **30** | 0 | 1 | 2 |
| **31** | 0 | 0 | 1 |
| **PSC** |  |  |  |
| **1** | 0 | 2 | 2 |
| **2** | 0 | 1 | 3 |
| **3** | 0 | 0 | 3 |
| **4** | 0 | 2 | 2 |
| **5** | 0 | 3 | 3 |
| **6** | 0 | 0 | 2 |
| **7** | 1 | 2 | 2 |
| **8** | 0 | 2 | 2 |
| **9** | 1 | 0 | 2 |
| **VBDS** |  |  |  |
| **1** | 0 | 0 | 3 |
| **2** | 0 | 3 | 3 |
| **LBDO** |  |  |  |
| **1** | 0 | 3 | 3 |
| **2** | 0 | 3 | 3 |
| **3** | 0 | 3 | 3 |
| **4** | 0 | 3 | 3 |
| **5** | 1 | 1 | 1 |
| **6** | 0 | 1 | 1 |

HPC hepatic progenitor cell, PBC primary biliary cholangitis, PSC primary sclerosing cholangitis, VBDS vanishing bile duct syndrome, LBDO large bile duct obstruction
