# Supplementary figures and images for: Keratin 7 expression in hepatic cholestatic diseases
Source: Virchows Arch. 2021 Jul 27;479(4):815–24. doi: 10.1007/s00428-021-03152-z (PMC8516784; doi:10.1007/s00428-021-03152-z)

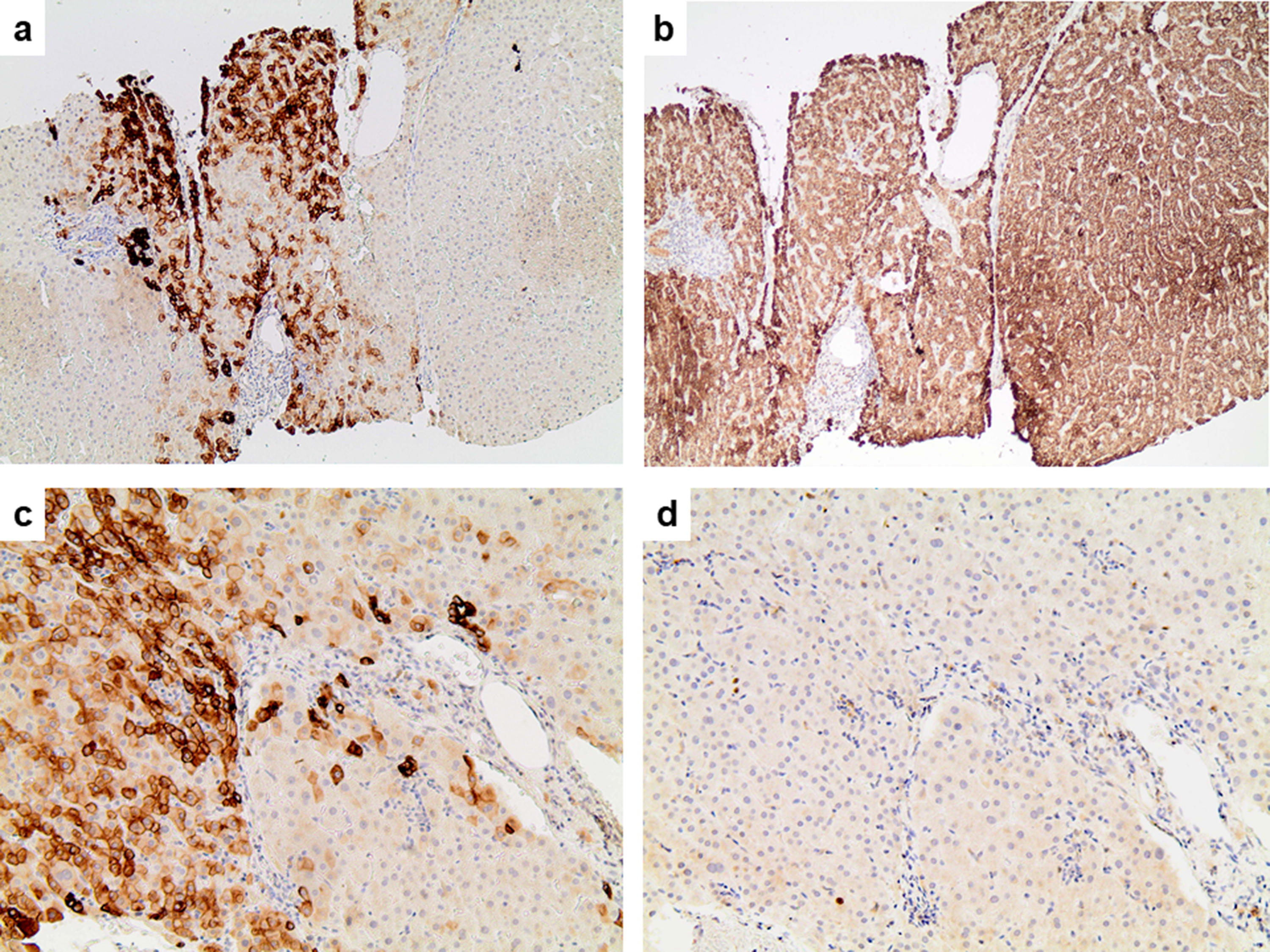

Supplement: Supplementary file 6 — a & c. The majority of hepatocytes around representative portal tracts are Keratin 7 (K7)-positive in a case of PBC where ≥50% of portal tracts had a similar appearance (Score 3); b. Serial section of 1a immunostained for HepPar1. All hepatocytes, including those expressing K7, show diffuse cytoplasmic granular HepPar1 immunostaining; d. Serial section of 1c immunostained for Ki-67. K7-positive hepatocytes are negative for Ki-67 indicating that they are resting cells (a, b x100, c, d x200 magnification). (PNG 8664 kb) [file 428_2021_3152_Fig3_ESM.png]

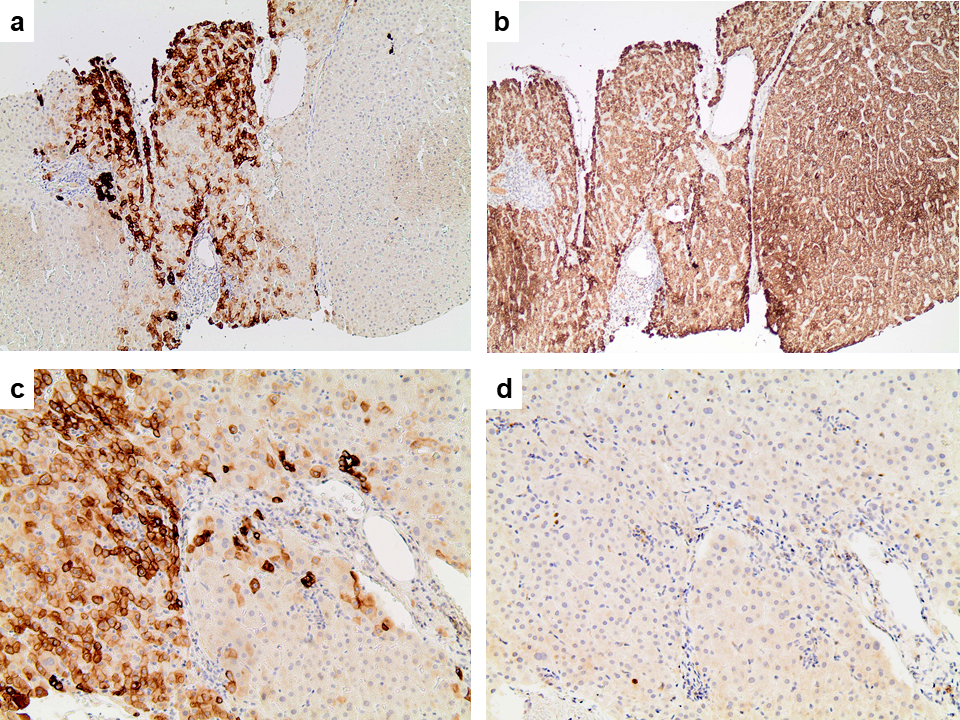

Supplement: Supplementary file 7 — High resolution image (TIF 1917 kb) [file 428_2021_3152_MOESM6_ESM.tif]

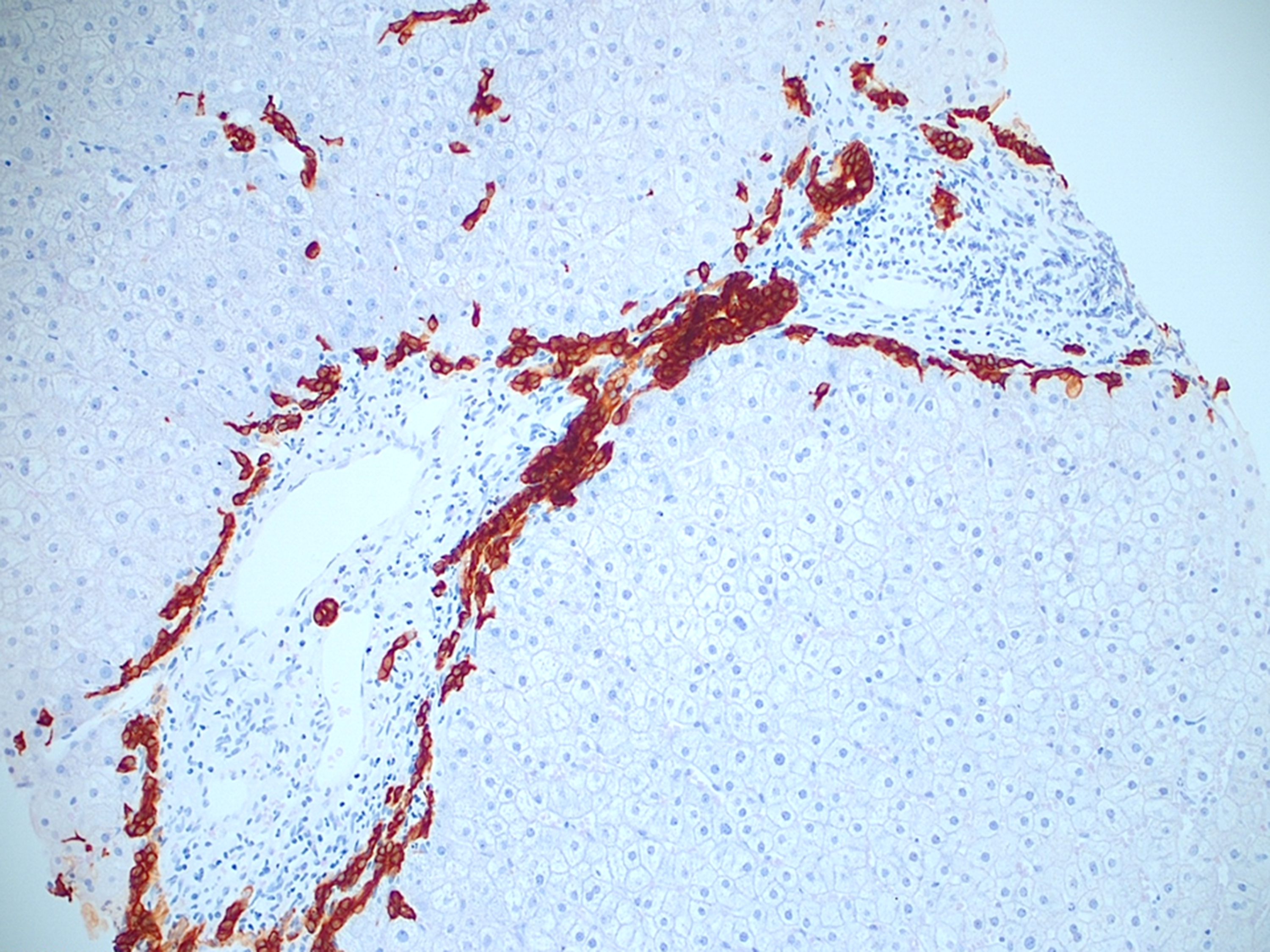

Supplement: Supplementary file 8 — Primary biliary cholangitis: Ductular reaction grade 3 and absence of K7-positive zone 1 hepatocyte expression (x100 magnification). (PNG 6737 kb) [file 428_2021_3152_Fig4_ESM.png]

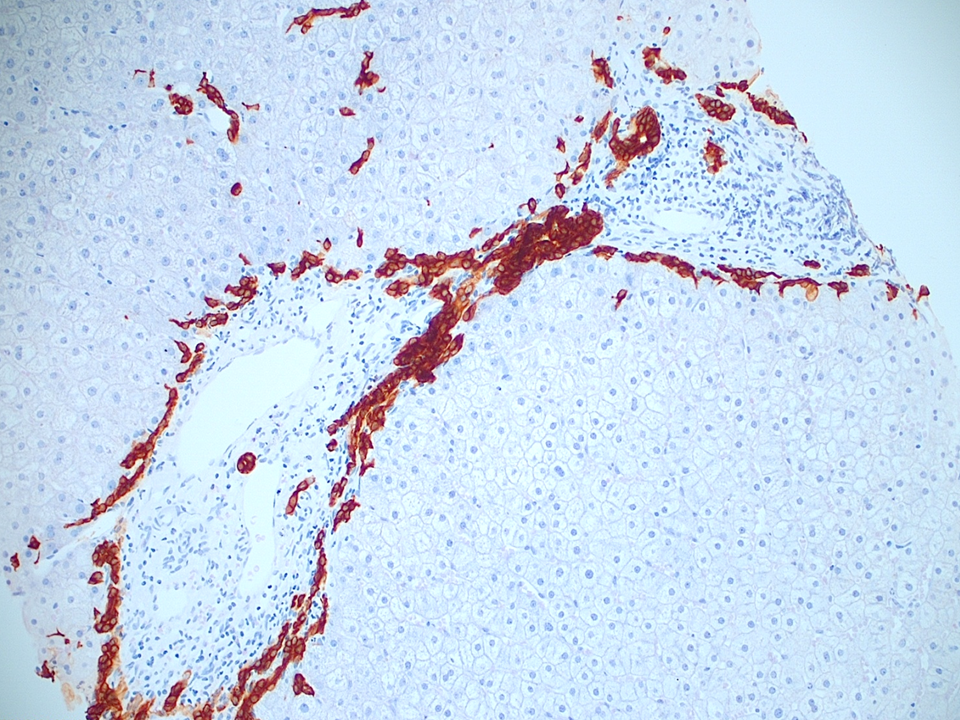

Supplement: Supplementary file 9 — High resolution image (TIF 1546 kb) [file 428_2021_3152_MOESM7_ESM.tif]

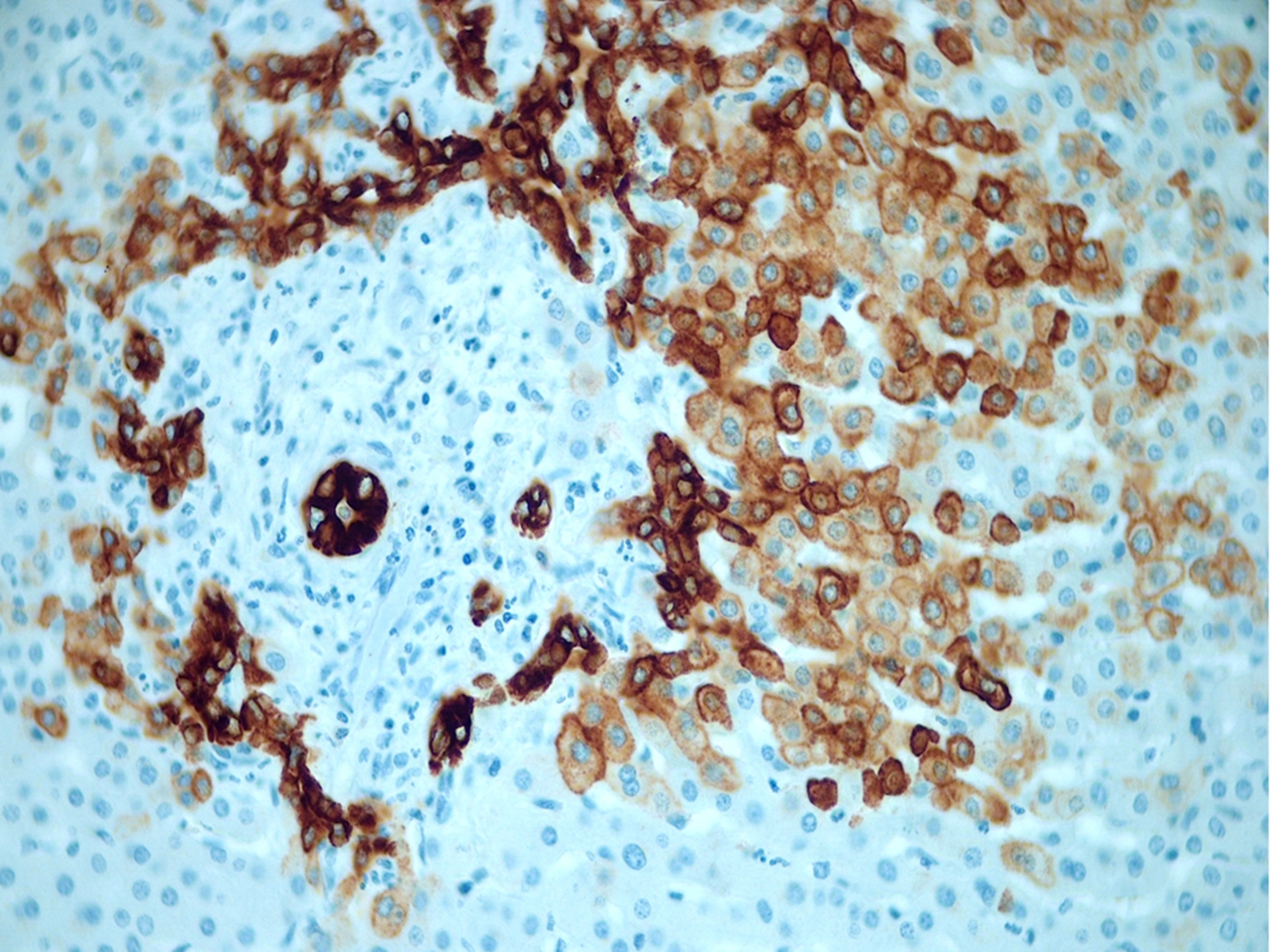

Supplement: Supplementary file 10 — Complete large bile duct obstruction: ductular reaction type 2A with score 3 K7-positive hepatocyte expression (x400 magnification). (PNG 6701 kb) [file 428_2021_3152_Fig5_ESM.png]

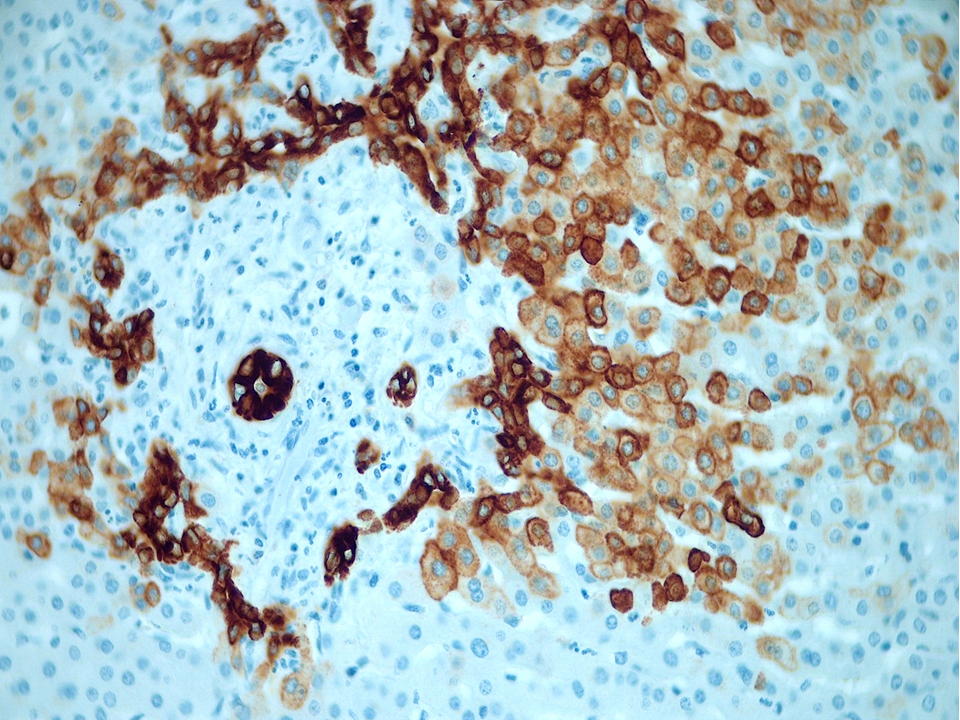

Supplement: Supplementary file 11 — High resolution image (TIF 1625 kb) [file 428_2021_3152_MOESM8_ESM.tif]
